# Identification of the *wbtF* gene as a cytotoxicity-associated factor in *Francisella novicida* infection

**DOI:** 10.3389/fcimb.2025.1647652

**Published:** 2025-10-09

**Authors:** Dhandy Koesoemo Wardhana, Takashi Shimizu, Kenta Watanabe, Akihiko Uda, Masahisa Watarai

**Affiliations:** ^1^ Laboratory of Veterinary Public Health, Joint Graduate School of Veterinary Medicine, Yamaguchi University, Yamaguchi, Japan; ^2^ Division of Veterinary Public Health, Department of Veterinary Science, Faculty of Veterinary Medicine, Universitas Airlangga, Surabaya, Indonesia; ^3^ Department of Veterinary Science, National Institute of Infectious Diseases, Tokyo, Japan

**Keywords:** *F. novicida*, *wbtF*, cytotoxicity, intracellular replication, tularemia

## Abstract

**Background:**

*Francisella tularensis* is a highly infectious Gram-negative bacterium that causes tularemia in humans and animals. It has a remarkable ability to survive and replicate within a wide range of host cells. *F. novicida* shares many characteristics with of *F. tularensis*. However, it is rarely pathogenic in humans, and its reduced virulence makes it a suitable model organism for studying *F. tularensis* infection. This study aimed to identify the pathogenic factors of *F. novicida*.

**Methods:**

Using a novel infection model with HeLa cells expressing FcγRII (HeLa-FcγRII cells), we screened 2,232 transposon mutants of *F. novicida* pre-treated with antiserum containing *F. novicida* antibodies to find less cytotoxicity strains. The transposon insertion site was identified by sequencing, leading to the determination of the genes responsible for the attenuated cytotoxicity. Additionally, the intracellular behavior of the mutant was investigated within both HeLa-FcγRII and THP-1 cells.

**Results and discussion:**

A total of thirteen mutants with attenuated cytotoxicity were isolated, and their responsible genes were identified. They are *figE, slt, fopA, iglC, igID, iglF, iglI, pdpB, pdpA, ampG*, *wbtF*, and one unnamed gene (FTN_0096). We focused on the *wbtF* gene. The *F. novicida* wild-type (WT) strain showed intracellular replication in HeLa-FcγRII and THP-1 cells, but the number of intracellular *wbtF* mutants decreased. The *wbtF* mutant could not escape from phagolysosomes in the initial phases of infection and was digested within the lysosome. The *wbtF* mutant was also detected in the mitochondria and the Golgi complex. The cytokine response induced by *wbtF* mutant was comparable to that of the WT strain. These findings indicate that *wbtF* is important for the intracellular replication of *F. novicida*.

## 1 Introduction

*Francisella tularensis* is a highly infectious Gram-negative bacterium that causes tularemia, a zoonosis affecting humans and animals. The genus *Francisella* comprises several species including *F. tularensis*, *F. philomiragia*, *F. noatunensis*, *F. hispaniensis* and *F. novicida* (Kingry and Petersen, 2014; Sjodin et al., 2012). *F. tularensis* is divided into two main subspecies: Type A (*F. tularensis subsp. tularensis* [*F. tularensis*]) and Type B (*F. tularensis subsp. holarctica* [*F. holarctica*]). Type A is considerably more virulent than Type B and is commonly associated with severe disease in humans, whereas Type B typically causes milder illness. Pneumonic tularemia, the most dangerous form, is transmitted through aerosols or inhalation. Remarkably, inhaling as few as 25 colony-forming units (CFUs) may result in pulmonary infection, making *F. tularensis* one of the most infectious bacteria transmitted via the aerosol route (Harrell et al., 2024). *Francisella* is a significant pathogen associated with high morbidity and mortality rates. The most virulent strain of *Francisella* induces systemic forms of tularemia (pneumonic, typhoidal), with fatality rates reaching 60% (Yeni et al., 2021; Wawszczak et al., 2022). Due to its high infectivity and potential for airborne spread, *F. tularensis* has been developed and stockpiled as a biological warfare agent by several nations (Dennis et al., 2001).

*F. novicida* is classified as a separate species from *F. tularensis*, but due to the high genomic homology, it is sometimes classified as a subspecies of *F. tularensis* (Johansson et al., 2010). Because *Francisella novicida* (*F. novicida*) is considerably less virulent than *F. tularensis*, it is commonly used as a surrogate strain for virulent *F. tularensis*. In contrast to *F. tularensis*, *F. novicida* is an opportunistic pathogen that causes illness and even death in debilitated or immunocompromised patients but not in healthy individuals. Due to the rarity of *F. novicida* infections, effective detection is challenging (Brett et al., 2012; Kingry and Petersen, 2014). *F. novicida* shares many characteristics with Type A strains of *F. tularensis*, showing similarities in the genome sequence, intracellular life cycle, and mechanisms of infectivity, including rapid phagosomal escape followed by vigorous cytosolic replication. It also infects macrophages, causing disease in mice. However, due to its lower virulence in humans, it can be safely handled under Biosafety Level 2 laboratory conditions (Kingry and Petersen, 2014; Marecic et al., 2017). *F. novicida* strain U112 possesses a potent tool in the form of a specific transposon mutant, which has been used to investigate the intracellular life cycle of *Francisella* and elucidate its virulence mechanisms (Weiss et al., 2007; Gallagher et al., 2007).

Typically, nonpathogenic bacteria taken up by host cells are enclosed in vacuoles or phagosomes, which mature through the endocytic pathway and ultimately fuse with lysosomes, leading to bacterial degradation. Intracellular pathogens have evolved strategies to evade this process within phagocytic cells and resist phagosome–lysosome fusion (Ray et al., 2009). In particular, *Francisella* species exhibit a complex intracellular life cycle and notable environmental persistence (Rowe and Huntley, 2015). Their survival and replication rely heavily on escaping the initial phagosomal compartment and replicating within the host cell cytosol, making *Francisella* a model cytosolic pathogen. The ability to persist and multiply inside phagocytes and other host cells is fundamental to its pathogenicity. Disruptions in key mechanisms, such as receptor engagement, resistance to reactive oxygen species (ROS), phagosomal escape, cytosolic replication, and evasion of innate immune recognition, significantly impair *F. tularensis* survival and reduce its virulence in both *in vitro* and *in vivo* models (Celli and Zahrt, 2013).

*wbtF* is assigned as a potential NAD-dependent epimerase and UDP-glucose 4 epimerase (UGE) (Thomas et al., 2007). It shows sequence similarity with established UGEs, including wbpP from *Pseudomonas aeruginosa* (Prior et al., 2003). wbpP is necessary for producing UDP-N-acetyl-D-galacturonic acid, a precursor to the galactosaminuronic acid-derived components of the O-antigen (Miller et al., 2008). UGE, part of the short-chain dehydrogenase/reductase enzyme superfamily, catalyzes the final step in the interconversion of UDP-glucose and UDP-galactose during galactose metabolism in bacteria and mammals (Fushinobu, 2021). Thomas et al. (2007) identified *wbtF* genes as a virulence determinant of *F. tularensis*, but its function remains unknown.

In this study, we created a transposon mutant library of *F. novicida* and screened for infection using epithelial cells, HeLa cells expressing the FcγRII (HeLa-FcγRII cells) (Nakamura et al., 2024). We focused our investigation on *wbtF* and analyzed that the *wbtF* was associated with the intracellular replication of *F. novicida*.

## 2 Materials and methods

### 2.1 Bacterial strains and culture conditions

*F. novicida* U112 was obtained from the Pathogenic Microorganism Genetic Resource Stock Center (Gifu University) and cultured aerobically at 37°C in a chemically defined medium (CDM) (Nagle et al., 1960) or on brain–heart infusion broth enriched with cysteine (BHIc) (McGann et al., 2010) and solidified with 1.5% agar (Wako Laboratory Chemicals, Osaka, Japan).

### 2.2 Cell culture conditions

HeLa cells were maintained in Dulbecco’s Modified Eagle Medium (DMEM; Sigma-Aldrich, St. Louis, MO, USA) supplemented with 10% heat-inactivated fetal bovine serum (FBS; Thermo Fisher, Waltham, MA, USA). The cultures were incubated at 37°C in a humidified atmosphere containing 5% CO_2_. Similarly, the human monocytic cell line THP-1 was cultured under the same incubation conditions in RPMI 1640 medium (Sigma-Aldrich, St. Louis, MO) supplemented with 10% heat-inactivated FBS.

### 2.3 Transposon mutant library construction

A transposon mutant library was constructed using the Ez-Tn5 Transposome system (Epicentre, Madison, WI, USA). The multiple cloning sites of plasmid pMOD3 were digested with *Hin*dIII and *Eco*RI to construct the transposon. A kanamycin resistance cassette from pKEK1140 (Rodriguez et al., 2008) was inserted into these sites, creating the plasmid pMOD3-FtKm. The transposon region of pMOD3-FtKm was amplified by polymerase chain reaction, purified, and combined with transposase according to the manufacturer’s instructions. This transposon mixture was introduced into *F. novicida* by cryotransformation. Following transformation, the bacteria were plated on BHIc agar supplemented with 50 μg/mL kanamycin to select for mutants.

### 2.4 Cytotoxicity assay

HeLa-FcγRII cells (5 × 10^4^ cells/well) were seeded in 48-well tissue culture plates and incubated for 24 h. *F. novicida* strains preincubated with mouse serum containing anti-*F. novicida* antibodies were added at a multiplicity of infection (MOI) of 1. The plates were centrifuged at 300 ×*g* at room temperature for 10 min to promote bacterial contact with the cells and then incubated at 37°C for the designated times. Following incubation, the cells were washed three times with DMEM to remove nonadherent bacteria and treated with gentamicin (50 μg/mL) for 1 h to eliminate extracellular bacteria. Infected cells were monitored microscopically at designated time intervals to assess the relative cytotoxicity of the transposon mutant library. To evaluate cytotoxicity, lactate dehydrogenase (LDH) release was measured after incubating cells in DMEM at 37°C for a defined period. LDH levels in the culture supernatant were then quantified using the LDH Cytotoxicity Detection Kit (Takara Bio, Shiga, Japan). In brief, cell culture supernatants were collected and incubated with the catalyst and dye solutions for 30 min at room temperature in the dark. Absorbance was measured at 490 nm with a reference wavelength of 600 nm using a microplate reader. LDH activity was expressed as a percentage of total LDH release, determined from lysed cell controls.

### 2.5 Sequence analysis of transposon mutants

The plasmid pMOD3 carries the R6Kγ origin of replication, facilitating propagation in *Escherichia coli*. Genomic DNA from *F. novicida* transposon mutants was extracted using a PureLink Genomic DNA Mini Kit (Thermo Fisher) and digested with restriction enzymes (*Xho*I, *Bgl*II, *Eco*RI, *Sal*I, *Not*I, *Bam*HI, *Pst*I, and *Sph*I). The resultant DNA fragments were blunted using a DNA Blunting Kit (Takara Bio) and then ligated using Ligation High Ver. 2 (Toyobo). The ligation products were introduced into One Shot PIR1 Chemically Competent *E. coli* (Thermo Fisher) via transformation. Kanamycin-resistant colonies were selected, and plasmid DNA was isolated. Sequencing was performed using the primers specified in the Ez-Tn5 Transposome system manual to determine transposon insertion sites.

### 2.6 Green fluorescent protein–expression by *F*. *novicida* and complementary strains

GFP-expressing plasmids (pOM5-GFP) were generated as described by Shimizu et al. (2019). Briefly, the plasmids were introduced into the wild-type (WT) and *wbtF* mutant strains of *F. novicida* via electroporation. To create pOM5-wbtF or complementary strains, *wbtF* of *F. novicida*, along with its native promoter region (300 bp upstream), was cloned into the pOM5 vector. The resultant plasmid (pOM5-wbtF) was used to transform the *wbtF* mutant strains of *F. novicida* via electroporation for complementation studies.

### 2.7 Intracellular replication assay

THP-1 cells (4 × 10^5^ cells/well) were seeded in 24-well tissue culture plates and differentiated by treatment with 200 nM phorbol 12-myristate 13-acetate (PMA) for 48 h. HeLa-FcγRII cells (1 × 10^5^ cells/well) were cultured overnight on 12-mm glass coverslips placed in 24-well plates. *F. novicida* strains were added to the THP-1 cells and *F. novicida* strains were opsonized with mouse serum added to the HeLa-FcγRII cells at an MOI of 1. After infection, the THP-1 and HeLa-FcγRII cells were washed three times with RPMI 1640 medium and DMEM, respectively. To remove extracellular bacteria, all wells were treated with gentamicin (50 μg/mL) for 1 h. Then, the cells were incubated in fresh medium at 37°C for the designated times. To analyze intracellular replication, infected cells were washed with phosphate-buffered saline and lysed using 0.1% Triton X-100 in chemically defined medium (CDM). The lysates underwent serial dilution and were plated on BHIc agar to determine the number of CFUs.

### 2.8 Fluorescence microscopy

THP-1 cells (4 × 10^5^ cells/well) were seeded on 12-mm glass coverslips in 24-well tissue culture plates and differentiated with 100 nM phorbol 12-myristate 13-acetate (PMA) for 48 h. HeLa-FcγRII cells (1 × 10^5^ cells/well) were cultured overnight on 12-mm glass coverslips placed in 24-well plates. The THP-1 cells were infected with GFP-expressing *F. novicida* strains and incubated for the designated times. For HeLa-FcγRII cells, GFP-expressing *F. novicida* strains were preincubated with mouse serum and added at an MOI of 1, followed by incubation for the designated times. The cells were stained with LysoTracker Red DND-99, MitoTracker Deep Red FM, and BODIPY TR Ceramide (Thermo Fisher) to visualize lysosomes, mitochondria, and the Golgi complex, respectively. To detect lysosomal-associated membrane protein 1 (LAMP-1), cells were fixed using a PLP Solution Set (FUJIFILM Wako Chemicals) containing 5% sucrose for 1 h at 37°C and then permeabilized using ice-cold methanol for 10 s. The fixed cells were incubated with anti-LAMP-1 antibody (ab25245, 1:100; Abcam), followed by staining with TRITC-conjugated anti-rat IgG secondary antibody (ab150158, 1:1000; Abcam). Confocal images were captured using a FluoView FV1000 laser scanning microscope (Olympus, Tokyo, Japan).

### 2.9 ELISA

THP-1 cells (4 × 10^5^ cells per well) were seeded into a 48-well culture plate and treated with 100 nM PMA for 48 hours. Following this, they were infected with *F. novicida* strains, including the *wbtF* mutant and its complementary strains. After 2, 6, 12, and 24 h of incubation, the levels of tumor necrosis factor (TNF)-α and interleukin (IL)-6 in the culture supernatants were quantified using the ELISA MAX Standard Kit (Biolegend, San Diego, CA), following the manufacturer’s protocol.

### 2.10 Statistical analysis

Differences between groups were evaluated using Welch’s *t*-test or multiple comparison tests, including the Bonferroni and Dunnett methods, as appropriate. Before these analyses, data normality was assessed using the Shapiro–Wilk test. *P* < 0.01 was considered statistically significant.

## 3 Results

### 3.1 Thirteen genes in the *F. novicida* transposon mutant library showed less cytotoxicity to HeLa-FcγRII cells

**Figure 1:**
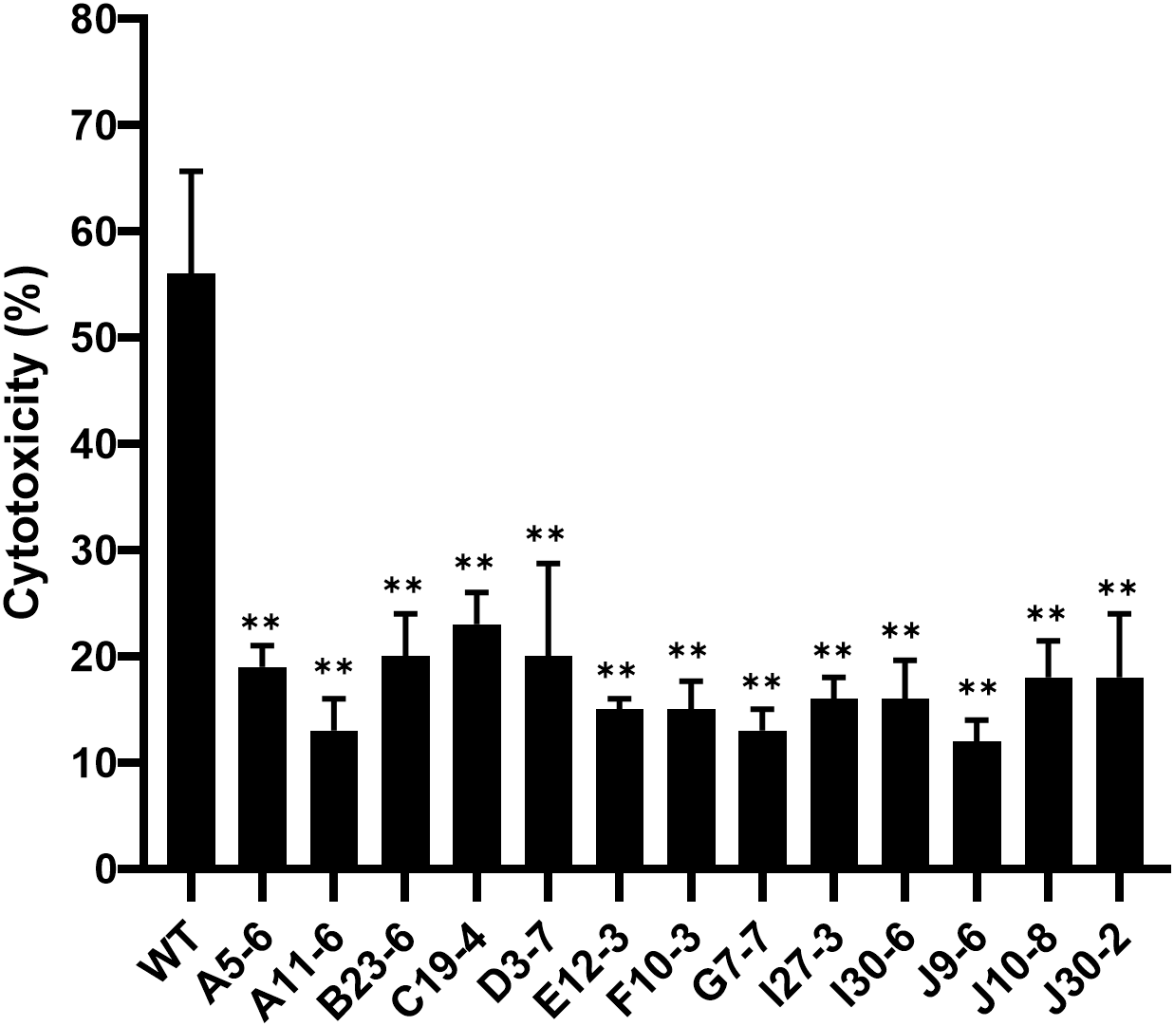
HeLa-FcγRII cells were infected with transposon mutants of *F. novicida* preincubated with mouse serum containing anti-*F. novicida* antibodies at an MOI of 1 and incubated at 37°C for 24 h. LDH release was measured as an indicator of cytotoxicity. The graph presents the mean and standard deviations (SD) of the LDH release at 24 h from three identical experiments. Statistical significance relative to the WT strain was determined using multiple comparisons and is indicated by asterisks. **P < 0.01.

We constructed an *F. novicida* transposon mutant library to identify the virulence factors of *F. novicida*. We previously developed a novel infection model of *Francisella* using HeLa cells expressing mouse FcγRII (HeLa-FcγRII) cells (Nakamura et al., 2024). In this cell line, cellular uptake occurs via antibodies and FcγRII. Therefore, it was expected that screening with this cell line would allow us to identify novel virulence factors crucial for pathogenesis after cellular uptake. We screened the *F. novicida* transposon mutant library using HeLa-FcγRII cells. *F. novicida* exhibits cytotoxicity toward epithelial cells, including HeLa-FcγRII cells, leading to cell death. We microscopically screened the transposon mutant library for strains lacking cytotoxic activity to identify genes associated with this cytotoxic effect. Of the 2,232 transposon mutants screened, 13 were identified as less cytotoxic. Significant differences in host cell condition were evident, while wild-type infection caused pronounced disruption and cell loss, monolayers exposed to the 13 mutants retained their structure and remained largely intact. To confirm these results, LDH assays were performed. The mutant strains caused less LDH release from HeLa-FcγRII cells than the WT strain, indicating reduced cytotoxicity (**Figure 1**). The insertion sites in these mutant strains were sequenced to determine the disrupted genes (**Table 1**). We focused on mutants with the code number G7-7, which encode *wbtF*, and analyzed the gene function.

**Table 1:** Sequence analysis of transposon mutant library.

Strain	Locus_tag	Gene name	Product
A5-6	FTN_0496	Slt	Soluble lytic transglycosylase
A11-6	FTN_0756	fopA	Outer membrane protein A
B23-6	FTN_1641	ampG	peptide-acetyl-coenzyme A transporter family protein
C19-4	FTN_0496	Slt	Soluble lytic transglycosylase
D3-7	FTN_1321	IglD	Intracellular growth locus protein D
E12-3	FTN_0096	–	Conserved hypothetical membrane protein
F10-3	FTN_1309	pdpA	Protein of unknown function
I27-3	FTN_1686	figE	Hypothetical membrane protein
G7-7	FTN_1425	wbtF	NAD dependent epimerase
I30-6	FTN_1322	IglC	Intracellular growth locus protein C
J9-6	FTN_1313	IglF	Hypotethical protein
J10-8	FTN_1317	IglI	Protein of unknown function
J30-2	FTN_1310	pdpB	Protein of unknown function

### 3.2 Effect of *wbtF* on intracellular replication in HeLa-FcγRII cells

**Figure 2:**
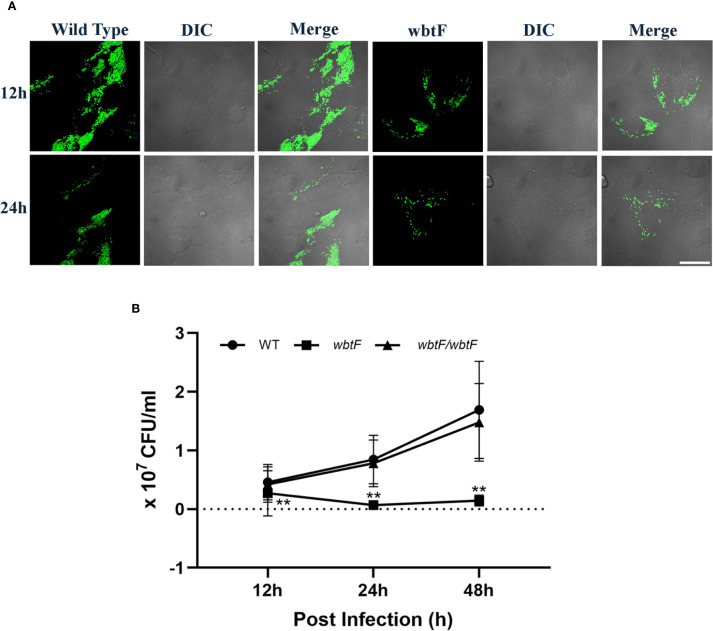
HeLa-FcγRII cells were infected with *F. novicida* strains preincubated with mouse serum containing anti-*F. novicida* antibodies at an MOI of 1, followed by gentamicin treatment (50 μg/mL) for 1 h **(A)** The infected HeLa-FcγRII cells were fixed and observed at 12 and 24 h postinfection. Scale bar = 20 μm. **(B)** At 12, 24, and 48 h postinfection, the infected HeLa-FcγRII cells were lysed with 0.1% Triton X-100, and the lysates were plated on BHIc agar. The graph presents the mean and SD of the number of CFUs at the designated time points from three identical experiments. Statistical significance relative to the WT strain at each designated time point was determined using multiple comparisons and is indicated by asterisks. **P < 0.01.

To determine the effect of *wbtF* on intracellular replication in HeLa-FcγRII cells in detail, we infected them with *F*. *novicida* WT and the *wbtF* mutant. Through a fluorescence microscope, we discovered intracellular bacteria using GFP-expressing *F. novicida* strains. We analyzed the intracellular of the WT and *wbtF* mutant 12h and 24h after infection in HeLa-FcγRII cells (**Figure 2A**). From 12 to 48 h postinfection, the rate of intracellular replication of the WT strain increased, while that of the *wbtF* mutant decreased (**Figure 2B**). Complementation of the *wbtF* mutant restored replication to the WT level (**Figure 2B**). These results suggested *wbtF* involvement in the intracellular replication of *F. novicida* in HeLa-FcγRII cells.

### 3.3 Role of *wbtF* in avoiding phagolysosomes in HeLa-FcγRII cells

**Figure 3:**
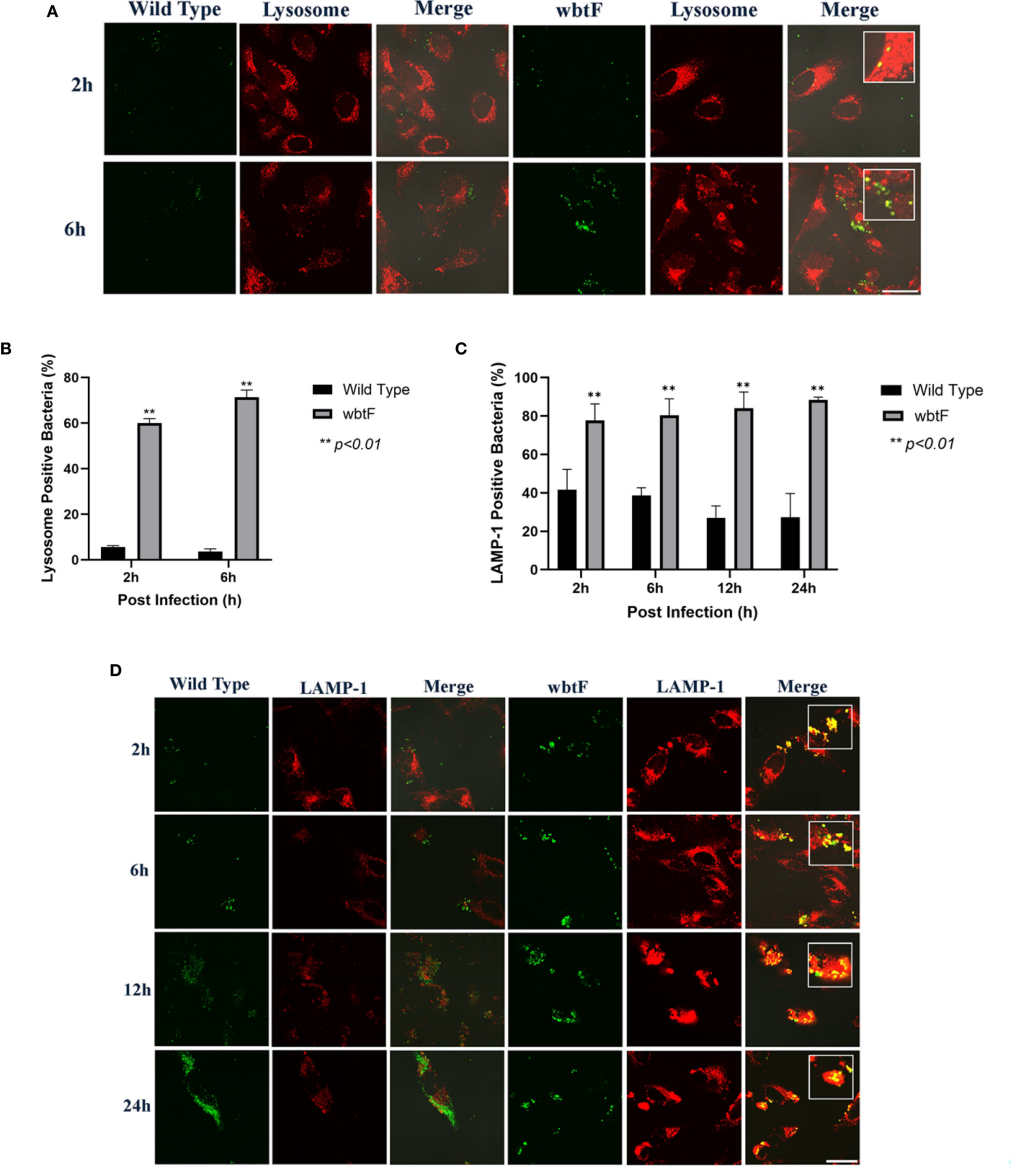
**(A)** HeLa-FcγRII cells were infected with *F. novicida* strains preincubated with mouse serum containing anti-*F. novicida* antibodies at an MOI of 1, followed by gentamicin treatment (50 μg/mL) for 1 h to eliminate extracellular bacteria. The infected HeLa-FcγRII cells were treated with LysoTracker Red DND-99 to visualize the acidic compartments at 2 and 6 h postinfection. Scale bar = 20 μm. **(B)** Percentage of *F. novicida* colocalization with LysoTracker-labeled lysosomes. Data represent the mean and SD from three identical experiments. Statistical significance relative to the WT strain at each designated time point was determined using Welch’s *t*-test and is indicated by asterisks. **P < 0.01. **(C)** Percentage of colocalization between *F. novicida* and LAMP-1. Data represent the mean and SD from three identical experiments. Statistical significance relative to the WT strain at each designated time point was determined using Welch’s *t*-test and is indicated by asterisks. **P < 0.01. **(D)** HeLa-FcγRII cells were similarly infected then treated with an anti-LAMP-1 antibody and stained with a TRITC-conjugated anti-rat IgG for 2–24 h postinfection. Scale bar = 20 μm.

We studied how the *wbtF* mutant replicates intracellularly in HeLa-FcγRII cells to clarify the role of *wbtF* in the intracellular replication of *Francisella* and its mechanism of escape from phagolysosomal digestion during the early stages of infection. HeLa-FcγRII cells were infected with GFP-expressing *F. novicida* strains. At 2 and 6 h postinfection, the cells were stained with LysoTracker and examined using confocal microscopy. The *F. novicida* WT bacteria did not exhibit LysoTracker colocalization. In contrast, the *wbtF* mutant strain exhibited noticeable colocalization with LysoTracker-positive compartments, suggesting defective phagolysosomal evasion (**Figure 3A**). A low percentage of WT bacteria showed lysosomal colocalization in HeLa-FcγRII cells. In contrast, a significantly higher percentage of *wbtF* mutant bacteria showed lysosomal colocalization (**Figure 3B**). This suggested that the WT bacteria, but not the *wbtF* mutant bacteria, escaped from the phagolysosomes in HeLa-FcγRII cells.

### 3.4 *F. novicida* escape from lysosomes in HeLa-FcγRII cells

HeLa-FcγRII cells were infected with GFP-expressing *F. novicida* WT and *wbtF* mutant strains to determine whether they underwent lysosomal digestion. Lysosomes were visualized using an anti-LAMP-1 antibody (**Figure 3D**). At 2–24 h postinfection, the WT strain replicated intracellularly, with bacterial numbers increasing over time. The *wbtF* mutant showed intracellular replication for up to 12 h postinfection. However, replication was decreased at 24 h postinfection, suggesting impaired long-term survival or replication within the host cells (**Figure 3C**). Compared with the *wbtF* mutant, a significantly lower percentage of WT strains that infected the HeLa-FcγRII cells colocalized with LAMP-1 at 2, 6, 12, and 24 h postinfection (**Figure 3C**). These results indicated that *F*. *novicida* WT escaped lysosomal degradation in the HeLa-FcγRII cells compared to the *wbtF* mutant, which exhibits reduced escape capacity.

### 3.5 Impact of *wbtF* in the mitochondria of HeLa-FcγRII cells

**Figure 4:**
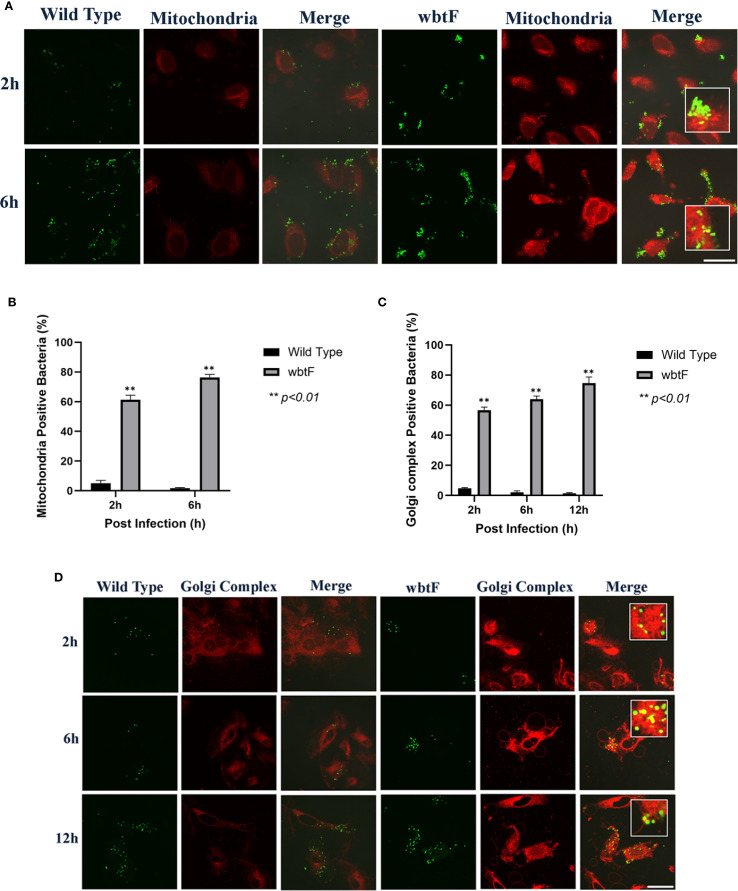
HeLa-FcγRII cells were infected with *F. novicida* strains preincubated with mouse serum containing anti-*F. novicida* antibodies at an MOI of 1, followed by gentamicin treatment (50 μg/mL) for 1 h to eliminate extracellular bacteria. **(A)** The infected HeLa-FcγRII cells were stained with MitoTracker Deep Red FM at 2 and 6 h postinfection. Scale bar = 20 μm. **(B)** Percentage of *F. novicida* colocalized with the mitochondria. Data represent the mean and SD from three identical experiments. Statistical significance relative to the WT strain at each designated time point was determined using Welch’s *t*-test and is indicated by asterisks. **P < 0.01. **(C)** Percentage of *F. novicida* colocalized with the Golgi complex. Data represent the mean and SD from three identical experiments. Statistical significance relative to the WT strain at each designated time point was determined using Welch’s *t*-test and is indicated by asterisks. **P < 0.01. **(D)** The infected HeLa-FcγRII cells were stained with BODIPY TR ceramide at 2, 6, and 12 h postinfection. Scale bar = 20 μm.

We infected HeLa-FcγRII cells with WT and *wbtF* mutant strains to determine the role of *wbtF* in the mitochondria. We visualized the mitochondria using MitoTracker Deep Red FM (**Figure 4A**) to observe whether the WT and the *wbtF* mutant strains colocalized with the mitochondria. At 2 and 6 h postinfection, a low percentage of the HeLa-FcγRII cells infected with the WT strain colocalized with the mitochondria (**Figure 4B**). In contrast, HeLa-FcγRII cells infected with the *wbtF* mutant showed 61%–76% colocalization with the mitochondria (**Figure 4B**). These results demonstrated that *F*. *novicida* WT effectively modified mitochondrial function and sustained replication, while the *wbtF* mutant could not.

### 3.6 Effect of *wbtF* on the Golgi complex of infected HeLa-FcγRII cells

We infected HeLa-FcγRII cells with WT and *wbtF* mutant strains to investigate the function of *wbtF* in the Golgi complex of infected cells. The Golgi complex was stained using BODIPY TR ceramide at 2, 6, and 12 h postinfection. A low percentage of WT bacteria colocalized with the Golgi complex (**Figure 4C**). In contrast, 57%–75% of the *wbtF* mutant bacteria colocalized with the Golgi complex at 2, 6, and 12 h postinfection (**Figure 4C**). Visualization of intracellular colocalization of the bacteria with the Golgi complex 2, 6, and 12 h postinfection can be seen in **Figure 4D**.

### 3.7 Effect of *wbtF* on intracellular replication in THP-1 cells

**Figure 5:**
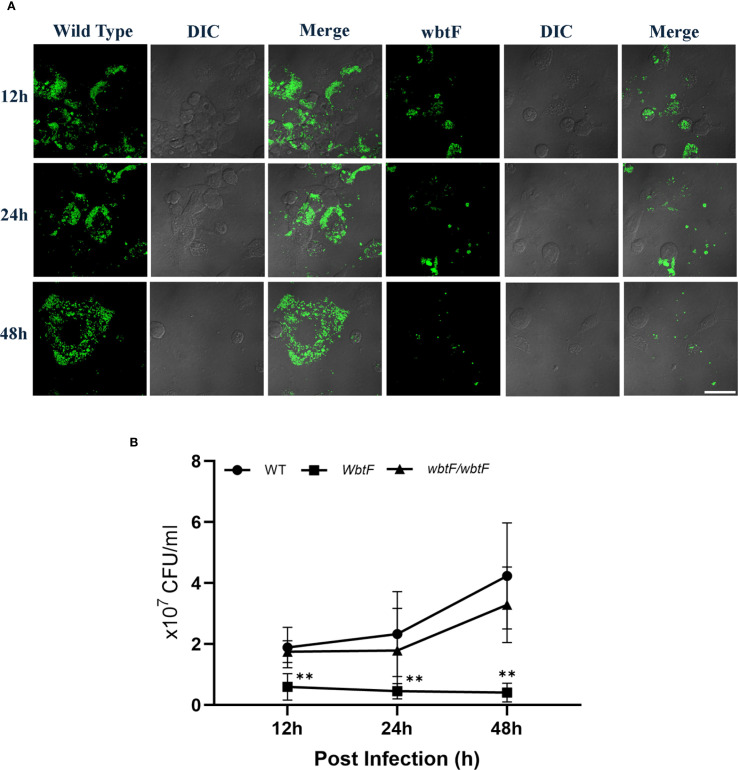
THP-1 cells were infected with *F. novicida* at an MOI of 1, followed by gentamicin treatment (50 μg/mL) for 1 h to eliminate extracellular bacteria. **(A)** The infected THP-1 cells were fixed and observed at 12, 24, and 48 h after infection. Scale bar = 20 μm. **(B)** The infected THP-1 cells were lysed with 0.1% Triton X-100 at 12, 24, and 48 h postinfection, and the lysates were plated on BHIc agar. Data represent the mean and SD from three identical experiments. Statistical significance relative to the WT strain at each designated time point was determined using multiple comparisons and is indicated by asterisks. **P < 0.01.

THP-1 cells were infected with the WT and *wbtF* mutant of *F. novicida* to evaluate the role of *wbtF* in intracellular replication. Fluorescence microscopy using GFP-expressing strains confirmed the presence and localization of intracellular bacteria. Our analysis of the intracellular levels of WT and *wbtF* mutant bacteria at 12, 24, and 48 h postinfection in THP-1 cells further validated the reduced replication capacity of the *wbtF* mutant compared with the WT strain (**Figure 5A**). The WT strain showed a steady increase in intracellular replication, while the number of *wbtF* mutants was significantly decreased between 12 and 48 h postinfection (**Figure 5B**). Complementation of the *wbtF* mutant restored replication to the WT level, confirming the role of *wbtF* in intracellular survival (**Figure 5B**). These findings suggested *wbtF* involvement in *F. novicida* intracellular replication in THP-1 cells.

### 3.8 Role of *wbtF* in avoiding phagolysosomes in THP-1 cells

**Figure 6:**
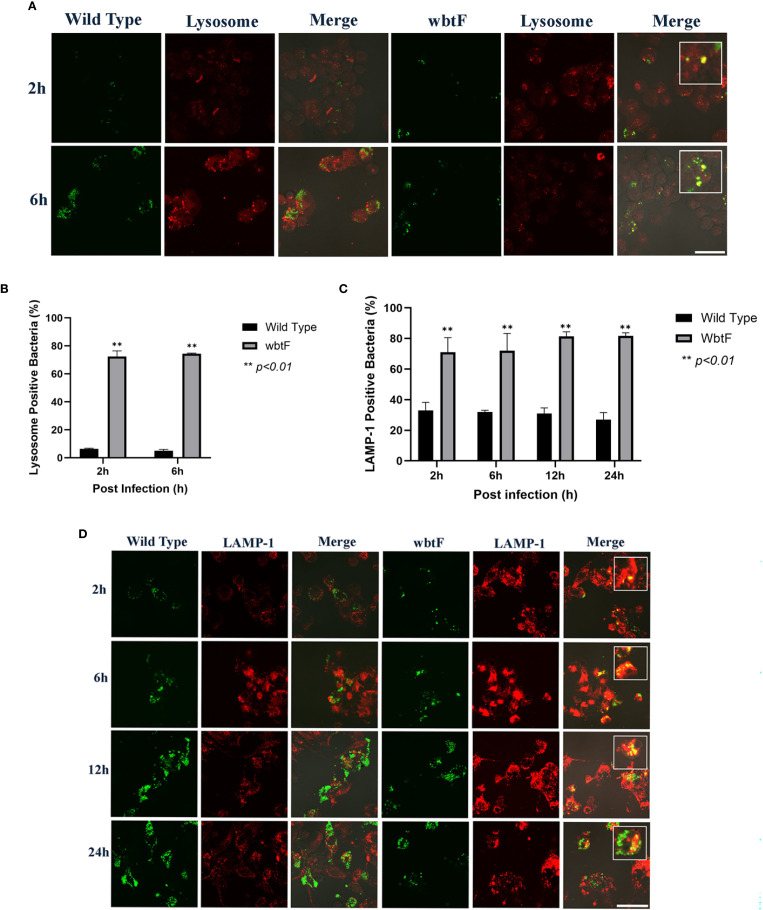
THP-1 cells were infected with *F. novicida* strains at an MOI of 1, followed by gentamicin treatment (50 μg/mL) for 1 h to eliminate extracellular bacteria. **(A)** The infected THP-1 cells were treated with LysoTracker Red DND-99 for 2–6 h postinfection. Scale bar = 20 μm. **(B)** Percentage of *F. novicida* colocalized with lysosomes. Data represent the mean and SD from three identical experiments. Statistical significance relative to the WT strain at each designated time point was determined using Welch’s *t*-test and is indicated by asterisks. **P < 0.01. **(C)** Percentage of *F. novicida* colocalized with LAMP-1. Data represent the mean and SD from three identical experiments. Statistical significance relative to the WT strain at each designated time point was determined using Welch’s *t*-test and is indicated by asterisks. **P < 0.01. **(D)** The infected THP-1 cells were treated with anti-LAMP-1 antibody and stained with TRITC-conjugated anti-rat IgG for 2–24 h postinfection. Scale bar = 20 μm.

We infected THP-1 cells with GFP-expressing *F. novicida* strains to investigate the role of *wbtF* in intracellular survival, specifically its involvement in phagolysosomal escape. We assessed the ability of *F. novicida* to evade phagolysosomal fusion during the initial stage of infection (2 and 6 h postinfection) using LysoTracker staining visualized under confocal microscopy. *F. novicida* WT showed low levels of colocalization with LysoTracker, indicating efficient evasion of phagolysosomal fusion. In contrast, the *wbtF* mutant colocalized with LysoTracker-positive lysosomes (**Figure 6A**). Only a limited number of WT bacteria were colocalized with the lysosomes, while a significantly higher number of *wbtF* mutant bacteria were detected within the lysosomal compartments (**Figure 6B**). This suggested that the WT strain escaped from the phagolysosomes in THP-1 cells, but the *wbtF* mutant could not.

### 3.9 *F. novicida* escape from lysosomes in THP-1 cells

THP-1 cells were infected with the GFP-expressing *F. novicida* WT and *wbtF* mutant strains to investigate whether lysosomes contributed to *F. novicida* degradation. Lysosomes were visualized using an anti-LAMP-1 antibody (**Figure 6D**). The WT strain exhibited consistent intracellular replication at 2–24 h postinfection, as indicated by the increasing number of bacteria. In contrast, the *wbtF* mutant replicated up to 12 h postinfection, but the intracellular bacterial load declined markedly by 24 h postinfection. Compared with the *wbtF* mutant, a significantly lower percentage of WT strains colocalized with LAMP-1 at 2, 6, 12, and 24 h postinfection (**Figure 6C**). These results indicated that *F*. *novicida* WT escaped lysosomal degradation in the THP-1 cells, whereas the *wbtF* mutant could not.

### 3.10 Impact of *wbtF* in the mitochondria of THP-1 cells

**Figure 7:**
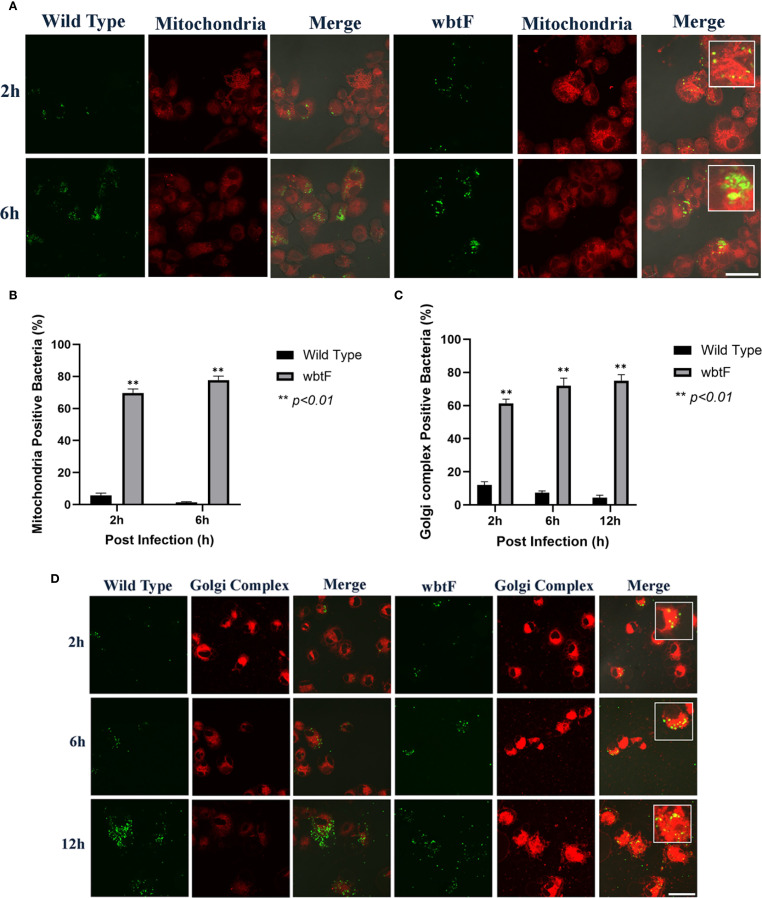
THP-1 cells were infected with *F. novicida* at an MOI of 1, followed by gentamicin treatment (50 μg/mL) for 1 h to eliminate extracellular bacteria. **(A)** The infected THP-1 cells were treated with MitoTracker Deep Red FM at 2 and 6 h postinfection. Scale bar = 20 μm. **(B)** Percentage of *F. novicida* colocalized with the mitochondria. Data represent the mean and SD from three identical experiments. Statistical significance relative to the WT strain at each designated time point was determined using Welch’s *t*-test and is indicated by asterisks. **P < 0.01. **(C)** Percentage of *F. novicida* colocalized with the Golgi complex. Data represent the mean and SD from three identical experiments. Statistical significance relative to the WT strain at each designated time point was determined using Welch’s *t*-test and is indicated by asterisks. **P < 0.01. **(D)** The infected THP-1 cells were treated with BODIPY TR ceramide at 2, 6, and 12 h after infection. Scale bar = 20 μm.

THP-1 cells were infected with WT and *wbtF* mutant strains to determine the role of *wbtF* in the mitochondria. We visualized the mitochondria using MitoTracker Deep Red FM (**Figure 7A**) to observe whether the WT and the *wbtF* mutant bacteria colocalized with the mitochondria. A low percentage of the WT bacteria colocalized with the mitochondria (**Figure 7B**). In contrast, 69%–78% of the *wbtF* mutant bacteria colocalized with the mitochondria at 2 and 6 h postinfection (**Figure 7B**). These results indicated that *F*. *novicida* WT modified mitochondrial function and sustained replication in the THP-1 cells, while the *wbtF* mutant could not.

### 3.11 Effect of *wbtF* on the Golgi complex of infected THP-1 cells

We infected THP-1 cells with the WT and *wbtF* mutant strains to analyze the function of *wbtF* in the Golgi complex. The Golgi complex was stained using BODIPY TR ceramide to determine if the Golgi complex could colocalize the WT strain and the *wbtF* mutant. The intracellular bacteria were examined at 2, 6, and 12 h after infection. A low percentage of the WT strain colocalized with the Golgi complex (**Figure 7C**). In contrast, 61%–75% of the *wbtF* mutant bacteria were colocalized with the Golgi complex at 2, 6, and 12 h after infection (**Figure 7C**). Visualization of intracellular colocalization of the bacteria with the Golgi complex 2, 6, and 12 h postinfection can be seen in **Figure 7D**.

### 3.12 Effect of *wbtF* on cytokine production

**Figure 8:**
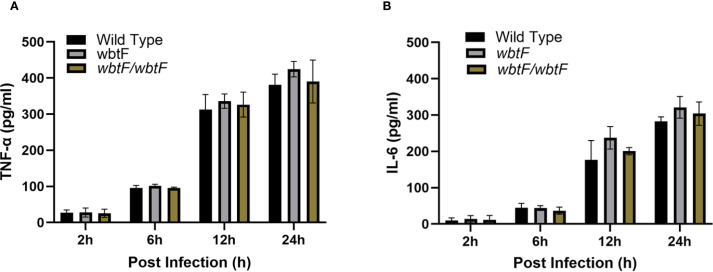
THP-1 cells were infected with *F. novicida* at an MOI of 1, followed by gentamicin treatment (50 μg/mL) for 1 h to eliminate extracellular bacteria. After 2 h, 6 h, 12 h, and 24 h of incubation, cell supernatants were collected, and the levels of TNF-α **(A)** and IL-6 **(B)** were determined using ELISA. The data represent the averages and standard deviations of three identical experiments. Statistical significance relative to the WT strain at each designated time point was determined using multiple comparisons and no significant difference was observed.

To evaluate the effect of *wbtF* in cytokine responses, we measured the induction of cytokines produced by THP-1 cells that were infected with WT, *wbtF* mutant and complementary strains. The induction of TNF-α (**Figure 8A**) and IL-6 (**Figure 8B**) did not differ significantly among the WT, *wbtF* mutant, and complementary strains.

## 4 Discussion

*Francisella* is a Gram-negative bacterium, and its highly pathogenic species, *F. tularensis*, is a well-known cause of tularemia in humans and other mammals. *F. tularensis* outmaneuvers the host’s immune defenses by escaping from the phagosome—a cellular structure meant to destroy invaders. Instead of being degraded, the bacteria multiply inside the host cell’s cytoplasm, where they are better protected from immune responses (Pavlik et al., 2024). Scientists are still piecing together the mechanism of phagosomal escape, and a gene cluster called the *Francisella* pathogenicity island (FPI) has emerged as a key player. The FPI encodes component proteins of a unique type VI secretion system thought to be critical to the bacterium’s survival and virulence (Clemens et al., 2015; Rigard et al., 2016; Spidlova and Stulik, 2017).

We constructed a transposon mutant library of *F*. *novicida* to isolate mutants that were less cytotoxic. We screened the transposon mutants using a new infection model of *Francisella*, HeLa cells expressing mouse FcγRII (HeLa-FcγRII). HeLa-FcγRII cells are a cell line that expresses the FcγRII receptor, enabling efficient uptake of IgG-opsonized bacteria (Nakamura et al., 2024). Although epithelial cells, like HeLa, are readily amenable to genetic modification, infection with *Francisella* is rarely effective and significantly lower than the infection efficiency of macrophage-like cells. *Francisella* infection of epithelial cell lines, such as like HeLa, A549, and HEK293, has been performed at a high MOI (100–10,000) and requires a relatively long time (approximately 8–24 h) (Lo et al., 2013; Amer et al., 2010; Bradburne et al., 2013). The ability of *Francisella* to infect macrophages largely depends on whether the bacteria are opsonized. The use of unopsonized bacteria leads to rapid disruption of the phagosome. In contrast, opsonizing *Francisella* with fresh serum facilitates directing the bacteria to various phagocytic receptors, resulting in delayed phagosomal escape (Geier and Celli, 2011). Notably, when HeLa cells engineered to express FcγRII were infected with antibody-coated bacteria, the infection efficiency was approximately 100 times higher compared with normal HeLa cells (Nakamura et al., 2024).

Among the 2,232 transposons mutants library, 13 showed less cytotoxicity and were successfully sequenced. Two mutants involved the same gene: FTN_0496 (*slt*). FTN_1686 (*figE*), FTN_0496 (*slt*), FTN_1322 (*IglC*), FTN_1321 (*IglD*), FTN_1309 (*pdpA*), FTN_1310 (*pdpB*), FTN_1317 (*IglI*), FTN_1313 (*IglF*), and FTN_0096 were previously reported as pathogenic factors of *Francisella* (Llewellyn et al., 2011; Ozanic et al., 2015; Brodmann et al., 2017; Pérard et al., 2018; Nakamura et al., 2019; Dean et al., 2020). FTN_0756 (*fopA*) and FTN_1641 (*ampG*) were previously reported as immunosuppressive factors involved in dampening host immune responses (Nakamura et al., 2022). However, this was the first report of FTN_1425 (*wbtF*). Therefore, we focused on *wbtF*. Although *wbtF* has been reported as a non-essential gene that can be disrupted (Thomas et al., 2007), we were unable remove the gene using a suicide vector (Nakamura et al., 2019) in this study. Therefore, we attempted to directly investigate *wbtF* gene from a transposon mutant library of *F. novicida*.

*wbtF* is one of the O-antigen biosynthetic gene clusters of *F. tularensis* (Clemens et al., 2012). The O-antigen capsule in *Francisella* comprises a polysaccharide that is structurally identical to the O-antigen found in lipopolysaccharide (LPS). This LPS O-antigen is a critical pathogenic factor. O-antigen deficiency results in increased sensitivity to serum and a loss of virulence in Type A and B strains of *F. tularensis* (Catanzaro and Inzana, 2020). Thomas et al. (2007) created knock-out mutants targeting the O-antigen gene cluster in *F. novicida* and *F. tularensis* SchuS4, leading to the complete loss of O-antigen production. Notably, the SchuS4 mutant lacking the O-antigen was fully attenuated in mice.

*Francisella* species are highly pathogenic, largely due to their ability to replicate within the cytosol of phagocytic cells, such as macrophages and dendritic cells. After entering these cells through phagocytosis, the bacteria are initially enclosed within a membrane-bound compartment called the *Francisella*-containing phagosome. However, they rapidly disrupt the phagosomal membrane and escape into the host cell cytosol, where they replicate. The bacteria adapt their metabolism to the cytosolic environment by relying on gluconeogenesis and amino acid catabolism as carbon and energy sources. Thus, phagosomal escape is critical for *Francisella*’s ability to replicate inside host cells, leading to disease *in vivo*. Phagosomal escape also triggers the host’s antimicrobial and innate immune defenses. In many cases, once the infected cell becomes overloaded with bacteria and dies, the bacteria exit and potentially spread to neighboring cells (Degabriel et al., 2023; Chong and Celli, 2010).

In this research, intracellular replication of the *F. novicida* WT strain in HeLa-FcγRII cells and THP-1 cells increased at 12–48 h after infection. This correlated with previous findings of increased intracellular replication of *Francisella* WT in HeLa-FcγRII and THP-1 cells (Nakamura et al., 2021, 2024). Compared with the WT strain, the *wbtF* mutant underwent intracellular replication for up to 12 h after infection in HeLa-FcγRII and THP-1 cells, but then decreased at 24 and 48 h after infection. The rate of intracellular replication of the *wbtF* mutant was significantly lower than that of the WT strain (*P <*0.01). Thomas et al. (2007) demonstrated that the O-antigen of *F. tularensis* was essential for the survival and replication inside host cells.

The acidic organelles were stained with LysoTracker. The *wbtF* mutant bacteria showed colocalization with LysoTracker in the early stages of infection (2–6 h) of HeLa-FcγRII cells (60%–71%) and THP-1 cells (72%–74%). Furthermore, the *wbtF* mutant also colocalized with the lysosome marker LAMP-1 at 2, 6, 12, and 48 h postinfection in 78%–88% of HeLa-FcγRII cells and 71%–82% of THP-1 cells.

*F. tularensis* virulence is closely associated with its ability to survive inside phagocytic cells and escape into the cytosol. Upon host cell entry, the bacterium is initially enclosed in a phagosome that typically matures into a phagolysosome. However, *F. tularensis* disrupts this maturation by blocking fusion between the phagosome and lysosome. Then, it breaks down the phagosomal membrane and escapes into the cytoplasm, where it replicates. The molecular mechanism underlying the phagosomal escape of *Francisella* remains poorly understood. However, it has been shown that disrupting genes within the FPI prevents the bacterium from phagosomal escape (Spidlova and Stulik, 2017). Thus, our findings suggest that the *wbtF* mutant could not escape from the phagosomes, indicating that *wbtF* is required for intracellular replication of *F*. *novicida*.

Mitochondria are double-membraned organelles vital for various cellular processes and cell survival. Their metabolic functions are crucial for maintaining tissue balance across different cell types and organisms. Unsurprisingly, these metabolic pathways often shift in response to stressors, like nutrient deprivation. Beyond their role in energy production, mitochondria are also highly involved in regulating cell death during infection and have been increasingly recognized as important hubs for immune signaling (Spinelli and Haigis, 2018; Mills et al., 2017). We observed that the *F. novicida* WT strain associates with mitochondria in HeLa–FcγRII cells and THP-1 cells, and underwent intracellular replication. Furthermore, it did not colocalize with mitochondria (MitoTracker) at 2–6 h after infection. In contrast, the *wbtF* mutant colocalized with the mitochondria during the initial stages of infection (2–6 h) in HeLa-FcγRII cells (61%–76%) and THP-1 cells (69%–78%).

Mitochondria are highly susceptible to damage, and their dysfunction disrupts cellular homeostasis. Such disturbances are closely linked to the development of various diseases, including neurodegenerative and cardiovascular conditions, cancer, metabolic disorders, and infections. Thus, the impaired mitochondria must be promptly isolated and selectively eliminated. Mitophagy (mitochondrial autophagy) is a critical cellular process that eliminates damaged mitochondria, thereby preserving mitochondrial and overall cellular homeostasis. When cells are exposed to stressors such as ROS, nutrient scarcity, or aging, mitochondria become depolarized and dysfunctional, triggering their selective removal through mitophagy (Doblado et al., 2021; Onishi et al., 2021; Xu et al., 2020; Lu et al., 2023).

Pathogens stimulate mitophagy to suppress the host defense mechanism. They likely employ a common mechanism that inhibits innate immune responses and promotes persistent infection by directly or indirectly facilitating mitophagy (Ma et al., 2024). The initiation of mitophagy to eradicate intracellular mitochondrial ROS (mtROS) is an essential survival tactic for intracellular infections. mtROS are essential for innate host defense, functioning as effectors by harming pathogens (Yang et al., 2025; Silwal et al., 2020). Alqahtani et al. (2023) showed that infection with *F. tularensis* live vaccine strain induced mitophagy. Generally, mitophagy is considered to limit the accumulation of mtROS, a significant source of cellular ROS. Microbial pathogens often evade or suppress the host’s protective mtROS pathway and neutralize bactericidal free radicals (Su et al., 2023; Silwal et al., 2020). Other intracellular bacteria, such as *Staphylococcus aureus*, promote mitophagy to clear mtROS. *S. aureus* not only survives within macrophages but also stimulates mitophagy in host cells to clear mtROS. It uses HDAC11 to augment interleukin 10 (IL-10) transcription, promoting mitophagy to clear mtROS, reduce mitochondrial damage, and maintain an ecological environment favorable for bacterial survival. *Listeria monocytogenes* diminishes mtROS and inhibits cell death by promoting mitophagy through the secretion of its virulence factor, listeriolysin O (Yang et al., 2025; Zhang et al., 2019). These findings suggest that the intracellular survival of *Francisella* WT depends on mitochondrial modulation and escape. Indeed, the effector protein *IglJ* has been reported to interact directly with mitochondrial targets (Prokšová et al., 2020). In contrast, the *wbtF* mutant cannot modulate mitochondria and is therefore detected in association with mitochondria. The wbtF gene might be involved in this kind of mitochondrial modulation.

The Golgi complex is a crucial membrane-bound organelle in the perinuclear region that consists of flattened disk-shaped cisternae connected in a ribbon-like structure. It comprises three distinct subcompartments: the *cis-*Golgi, medial-Golgi, and *trans*-Golgi network (Ahat et al., 2019). The Golgi complex is the modification and sorting hub for proteins and lipids. Newly synthesized proteins and lipids are delivered to the Golgi complex, where they undergo posttranslational modifications and are sorted into vesicles for transport to other organelles or extracellular secretion (Makhoul et al., 2019).

To explore the activities of the *F. novicida* WT and *wbtF* mutant strains within the Golgi complex, infected HeLa-FcγRII and THP-1 cells were stained with BODIPY TR ceramide. The *Francisella* WT strain only showed colocalization with the Golgi complex in 2%–5% of HeLa-FcγRII cells and 4%–12% of THP-1 cells. However, the *wbtF* mutant was colocalized with the Golgi complex in 57%–75% of HeLa-FcγRII cells and 61%–75% of THP-1 cells.

The Golgi complex is a crucial organelle in the host defense systems against intracellular infections. Additionally, recent research indicates that the Golgi complex plays a critical role in immunological signaling (Tao et al., 2020). Disruption of the structure of the Golgi complex catalyzes the pathogenesis of infectious diseases. Various intracellular bacterial, viral, and protozoan infections trigger Golgi complex destabilization. This evolutionarily conserved strategy enables pathogens to access nutrients, hijack organelle membranes for replication, and subvert host immune defenses (Read et al., 2024). Type I interferons (IFNs) are induced by the Golgi complex in response to various stimuli, including *Francisella* infection (Gregory et al., 2020). Type I IFNs are a group of cytokines crucial for shaping innate and adaptive immune responses during viral and bacterial infections. However, they also suppress antibacterial immunity by promoting IL-10 production, inhibiting proinflammatory cytokines and chemokines, and promoting immune cell apoptosis, thereby exacerbating *Francisella* infections (Xia et al., 2025; Li et al., 2024). The translocation of the signaling intermediate stimulator of IFN genes (STING, TMEM173) from the endoplasmic reticulum to the *cis*-Golgi is initiated by cyclic GMP-AMP synthase or interferon-γ inducible protein 16 following the identification of cytosolic exogenous DNA (Storek et al., 2015; Meunier et al., 2015; Man et al., 2015). STING activates TANK-binding kinase 1 (TBK1) at the Golgi complex by inducing its autophosphorylation at serine 172. Following activation, TBK1 phosphorylates interferon regulatory factor 3, facilitating dimerization and translocation to the nucleus, which drives the expression of immune-related genes, including type I IFNs (Barber, 2011). Interestingly, structural disruption of the Golgi complex using golgicide A blocks TBK1 phosphorylation in response to *F. tularensis* infection and LPS (Gregory et al., 2020). These results suggest that *F. novicida* WT escapes from the Golgi complex, but *wbtF*, which was detected in the Golgi complex, cannot escape from the immune response.

Since *wbtF* is a gene involved in O-antigen synthesis, we investigated the cytokine production of the *wbtF* deletion mutant to confirm whether the attenuated cytotoxicity and intracellular proliferation of the mutant were due to the LPS-mediated activation of immune responses. Infection of THP-1 cells with *F. novicida* WT, *wbtF* mutant, and the complemented strain produced similar TNF-α and IL-6 responses, indicating that *wbtF* does not significantly affect cytokine production. The *wbtF* gene, a consecutive gene located within the O-antigen-biosynthetic gene clusters spanning a 17-kb chromosomal region of *F. tularensis*, is directly implicated in LPS biosynthesis. The O-antigen constitutes one of the three components of LPS, along with the lipid A and the oligosaccharide core (Clemens et al., 2012; Krzyżewska-Dudek et al., 2024). *Francisella* LPS does not function as an antagonist of TLR4-mediated innate immune signaling in either humans or mice. *Francisella* LPS is tetraacylated, and it elicits only weak activation of TLR4 and demonstrates a markedly reduced ability to induce the secretion of inflammatory cytokines like TNF-α and IL-6 by murine and human innate immune cells (Hajjar et al., 2006; Gunn and Ernst, 2007; Noah et al., 2010). It seems that *Francisella* LPS represents, at most, a minor contributor to the induction of proinflammatory responses within the innate immune system. Although deletion of LPS synthesis genes, such as *kdsA*, have been reported to enhance cytokine production (Nakamura et al., 2022), the absence of *wbtF* did not impact cytokine production in this study. Taken together, these results imply that the attenuated cytotoxicity and intracellular growth ability observed in *wbtF* deletion mutant are likely due to mechanisms distinct from LPS-mediated immune activation. Although comprehensive understanding of the detailed mechanisms necessitates further investigation, our results may suggest that *wbtF* is important for modulating LPS structure, potentially facilitating the evasion of recognition and uptake by cellular organelles, including phagosomes, mitochondria, and the Golgi apparatus.

In summary, our research focused on identifying a virulence factor of *F. novicida* by screening a transposon mutant library. We used a new infection model of *Francisella* using HeLa cells expressing mouse FcγRII (HeLa-FcγRII) (Nakamura et al., 2024). We identified *wbtF* as a pathogenic factor of *F*. *novicida*. *wbtF* is required for escaping from organelles such as phagosomes, mitochondria, and the Golgi apparatus, followed by intracellular replication in HeLa-FcγRII and THP-1 cells. Thus, it may be a potential target for controlling *Francisella* infection. However, further research is necessary to elucidate the detailed mechanism of *wbtF* function in *Francisella* infection.

## Data Availability

The original contributions presented in the study are included in the article/supplementary material. Further inquiries can be directed to the corresponding author.
